# The Short Nicotine Dependence Index: A Simple and Versatile Self-Report Measure of Nicotine Dependence for General Populations

**DOI:** 10.1093/ntr/ntaf204

**Published:** 2025-10-08

**Authors:** Sarah E Jackson, Harry Tattan-Birch, John Stapleton, Martin J Jarvis

**Affiliations:** Department of Behavioural Science and Health, University College London, London, United Kingdom; Department of Behavioural Science and Health, University College London, London, United Kingdom; Independent Statistician; Department of Behavioural Science and Health, University College London, London, United Kingdom

## Abstract

**Introduction:**

Nicotine dependence measures often rely on self-reported cigarette consumption, which has declined over time and may not accurately reflect nicotine intake. We developed a brief two-item Short Nicotine Dependence Index (SNDI) assessing urge to use and difficulty abstaining, and examined its association relative to that of the established Heaviness of Smoking Index (HSI) with saliva cotinine, a biomarker of nicotine exposure.

**Methods:**

Data were drawn from the Health Survey for England (HSE; 2000–2021) and a London General Practice Survey (GP Survey; 1989) (*n* = 14 244 current cigarette smokers aged ≥16 with valid cotinine data). Dependence was assessed using two questions: time to first cigarette after waking (scored 1–6) and perceived difficulty going a whole day without smoking (scored 0–3). Scores were summed to produce a total SNDI score (range 1–9). Mean cotinine levels were estimated across item responses and total scores in each sample.

**Results:**

Cotinine concentrations increased consistently with higher scores on both individual items and the total SNDI score. In the HSE, cotinine ranged from 83 [95% CI = 77% to 88%] ng/mL for those with the lowest total score to 387 [377–396] ng/mL for those with the maximum score. A similar gradient was observed in the GP Survey (from 92 [75–110] to 431 [399–463]), despite higher overall cigarette consumption. In both datasets, the SNDI explained more variance in cotinine than the HSI: *R*^2^ = 0.304 vs. 0.278; GP Survey: 0.283 vs. 0.250.

**Conclusions:**

The SNDI is a brief self-report measure that outperforms existing short tools in predicting nicotine exposure. It offers a practical alternative for research and community surveillance in evolving nicotine use landscapes.

**Implications:**

The Short Nicotine Dependence Index offers an efficient and practical alternative to traditional dependence measures that rely on cigarette consumption. Its brevity and strong correlation with biochemical markers make it well-suited for use in large-scale surveys and clinical settings. By focusing on observed behavior (time to first cigarette) and self-reported difficulty abstaining, it remains relevant as smoking and nicotine use patterns shift. In addition, because it does not use reported cigarettes smoked per day, it may be useful for assessing dependence on other nicotine products (eg, e-cigarettes). Further research is needed to evaluate its validity among users of other nicotine products.

## Introduction

Despite declines in smoking prevalence,^1^ cigarette smoking continues to pose a major public health challenge, largely due to the persistent nature of nicotine dependence. Nicotine dependence is characterized by two key features: a strong urge or desire to use nicotine and difficulty abstaining.^2^ These aspects reflect both the rewarding effects of nicotine, which can create cravings, and the aversive effects of withdrawal, which make quitting difficult.^3^ Nicotine affects the brain’s reward system, reinforcing continued use over time and making it difficult for users to break the cycle, even when they recognize the harms or attempt to quit.^3^

Measuring nicotine dependence is essential for identifying individuals at risk of continued smoking, tailoring cessation treatments, and evaluating the effectiveness of interventions. It helps clinicians to provide support based on the severity of a person’s dependence, while also enabling researchers to better understand nicotine use patterns and the impact of different products. Accurate measurement is particularly important in public health and clinical research to track progress, inform policy, and improve outcomes in tobacco control efforts.

Several measures have been developed to assess nicotine dependence, each varying in complexity and focus. For example, the eight-item Fagerström Tolerance Scale (FTS), introduced in 1978, was one of the first attempts to quantify nicotine dependence, focusing on tolerance and withdrawal symptoms. However, researchers questioned its psychometric properties,^4^^,^^5^ highlighting flaws with some of the items that limit its usefulness, such as its use of a two-level item for “time to first cigarette”. In 1989, Heatherton et al.’s Heaviness of Smoking Index (HSI)^6^ refined the FTS by narrowing the focus to just two items: time to first cigarette and daily cigarette consumption. These were rescaled to offer greater sensitivity, with four levels for both. Later, in 1991, Heatherton revised the FTS, resulting in the Fagerström Test for Nicotine Dependence (FTND),^7^ which remains one of the most widely used tools for assessing nicotine dependence. The FTND improved upon the original scale by refining item scoring and including additional measures such as cigarette consumption and withdrawal symptoms.

Although these and other tools are widely used in research and clinical practice, a more streamlined and accessible measure would be useful for large-scale public health surveys and epidemiological studies.^8^ One key limitation of current tools is their reliance on cigarette consumption as an indicator of nicotine dependence, which presents several challenges. First, mean cigarette consumption has declined substantially over time,^9^^,^^10^ which means that categorizations developed in the 1980s may no longer be appropriate. For instance, the HSI defines its lowest category as fewer than 11 cigarettes per day,^6^ yet the current average in England falls below this threshold.^9^ As such, the index may no longer capture meaningful variation in dependence among people who smoke fewer cigarettes but still experience strong cravings or difficulty quitting. Supporting this, a 1999 paper suggested that the HSI and FTND captured little more than cigarette consumption in a sample with relatively low intake (mean = 12 cigarettes per day),^11^ suggesting these tools do not offer the most sensitive measure of dependence in modern contexts. In addition, a more recent paper reported that although mean cigarette consumption is declining, people are increasingly likely to smoke their first cigarette of the day within 30 minutes of waking, suggesting lower consumption does not necessarily reflect lower dependence.^12^ Second, cigarette consumption is prone to reporting bias, with people tending to round their reported consumption to convenient numbers, like 10, 15, or 20 cigarettes per day.^9^ Third, cigarette count does not reliably reflect nicotine intake. Data from both the National Health and Nutrition Examination Survey (NHANES) and the Health Survey for England (HSE) show that while daily cigarette consumption has declined, cotinine levels—a biomarker of nicotine exposure—have remained relatively stable.^13–15^ People can compensate for fewer cigarettes by smoking more intensively, such as by taking more puffs or inhaling more deeply, a behavior known as “titration”.^16^^,^^17^ As a result, reductions in cigarette consumption may not correspond to reduced nicotine or toxin exposure.^13–15^ Finally, nicotine use is no longer almost exclusively limited to cigarette smoking^18^; increased use of non-cigarette smoked tobacco^19^ and the rising prevalence of non-combustible nicotine products^20^ further limit the utility of consumption-based measures for capturing nicotine dependence in today’s more diverse nicotine landscape.

In this article, we propose a brief two-item self-report measure—the Short Nicotine Dependence Index (SNDI)—which captures the two key features of nicotine dependence: urge to use and difficulty abstaining. Using data from two surveys conducted at different points in time (1980s and 2000-2020s) with different levels of cigarette consumption, we examine its association with saliva cotinine concentration—a quantitative biomarker of nicotine intake that is a strong predictor of success in stopping smoking.^21^ We then compare its ability to account for variation in cotinine levels, as indicated by *R*^2^ values, to that of the widely used short index of nicotine dependence: the HSI.

## Materials and Methods

### Data Sources

#### Health Survey for England, 2000–2021

The HSE is an annual, nationally representative household survey in England, designed to monitor health trends and inform policy. Each year, the HSE includes questions on smoking behavior, cigarette consumption, and other relevant sociodemographic and health indicators. The survey uses a stratified, multistage probability sampling design and includes both interview and nurse visit components. Smoking status, time to first cigarette, and difficulty in abstaining for a whole day were assessed at the initial interview; while saliva specimens for cotinine were gathered at the nurse visit a week or so later. For our analyses, we used data from surveys conducted between 2000 and 2021 that assessed cotinine in participants aged ≥16 years (2000–2003, 2007–2011, 2013, 2015, 2017, 2019, and 2021).

#### South and West London General Practice Survey, 1989

The General Practice Survey (GP Survey) was a cross-sectional study conducted in 1989 across general practices in South and West London.^22^ It collected detailed information on smoking behaviors, including cigarette consumption and dependence indicators. Participants also provided saliva samples for assessment of cotinine. Although not nationally representative, this survey offers the opportunity to examine the association between our new dependence scale and cotinine concentrations in a different historical context.

### Participants

We analyzed data from participants aged ≥16 years who reported current cigarette smoking, had a valid cotinine measurement, and completed the two items assessing nicotine dependence. This provided a total sample of 14 244 participants; 12 736 from HSE and 1508 from the GP Survey.

### Measures

Cigarette smoking was assessed in the HSE with the question “Do you smoke cigarettes at all nowadays? (yes/no)” and in the GP Survey with the question “Do you smoke cigarettes?” with response options “yes”, “no, never been a cigarette smoker”, and “no, used to smoke but gave up”.

Nicotine dependence was assessed with two items. The first assessed time to first cigarette: “How soon after waking do you usually smoke your first cigarette of the day?” Response options were *less than 5 minutes* (scored 6), *5-14 minutes* (5), *15-29 minutes* (4), *30 minutes but less than 1 hour* (3), *1 hour but less than 2 hours* (2), and *2 hours or more* (1). These response options extended the four-level categorization used in the HSI and FTND (≤5, 6–30, 31–60, and >60 minutes).^6^^,^^7^ The second item assessed difficulty abstaining, with the question: “How easy or difficult would you find it to go without smoking for a whole day? Would you find it… very easy (scored 0), fairly easy (1), fairly difficult (2), or very difficult (3)?” For ease of use in practice, scores on the two items were summed to create a total SNDI score ranging from 1 to 9. This summation gives more weight to time to first cigarette due to its wider scoring range (1–6 versus 0–3). The scoring was designed to reflect the nature of each item: individuals who smoke more than 2 hours after waking may still show some degree of dependence, warranting a minimum score of 1, while those who find it very easy to abstain for a full day are unlikely to be dependent in any way, justifying a score of 0. In regression models predicting cotinine, both items showed similar independent associations with cotinine for each one-point increase (Supplementary File 1), supporting the use of a simple sum score.

Saliva cotinine concentration, a sensitive and specific marker of recent nicotine intake, was measured by the same laboratory in all the data presented here. In the 1989 GP survey and the HSE up to and including 2007, the method comprised liquid extraction and gas chromatography with nitrogen phosphorous detection, after which high-performance liquid chromatography coupled with mass spectrometry with multiple reaction monitoring was used (HSE 2008 onwards). The two methods were shown to be interchangeable in an across-laboratory validation study.^23^ Benowitz et al. have shown that daily nicotine intake in mg can be estimated by measured cotinine (ng/mL) in blood plasma or serum times a constant of 0.08.^24^^,^^25^ Since the concentration of cotinine in saliva is 25% higher than in blood,^26^ in our data this constant becomes 0.10. Thus, a saliva cotinine concentration of 100 ng/mL equates to an estimated daily intake of 10 mg nicotine.

Participants also reported their usual daily cigarette consumption, age, and sex. We used data on daily cigarette consumption (scored 0 for those reporting ≤10 cigarettes per day, 1 for 11–20, 2 for 21–30, and 3 for >30) and time to first cigarette (scored 0 for those responding ≥1 hour, 1 for 30–59 minutes, 2 for 5–29 minutes, and 3 for <5 minutes) to calculate HSI scores ranging from 0 to 6.^6^ It was not possible to calculate FTND scores because data were not collected on all of the necessary items.

### Statistical Analysis

Data were analyzed using SPSS version 29. Analyses of HSE data were weighted to account for the complex sampling design.

Within each survey, we report descriptive data on sample characteristics (age, gender, cigarette consumption, and cotinine) and the distribution of participants across levels of dependence. We estimated mean cotinine levels (with 95% confidence intervals [CI]) in relation to each SNDI item, separately and as a composite score. For context, we also report mean daily cigarette consumption in relation to each SNDI item and the composite score.

We tested the internal consistency of the two scale items within surveys using Cronbach’s alpha. We also ran a series of regression models predicting cotinine from each item and the composite score of the SNDI (each in separate models) and compared *R*^2^ values with equivalent models using the HSI.

## Results

We analyzed data from 12 736 participants in the HSE between 2000 and 2021 and 1508 in the GP Survey in 1989. Relative to the GP Survey sample, the HSE sample was slightly older on average (mean = 41.6 vs. 38.1 years), a higher proportion were women (49.5% vs. 37.2%), and they reported smoking fewer cigarettes per day (mean = 12.9 vs. 16.4; 4.0% vs. 1.3% reported smoking <1 cigarette per day, 4.1% vs. 2.9% 1–2 cigarettes, and 12.1% vs. 9.2% >2–5 cigarettes, 25.4% vs. 24.8% >5–10 cigarettes, 42.8% vs. 42.5% >10–20 cigarettes, 11.5% vs. 19.2% >20 cigarettes). However, average cotinines were broadly similar (274 ng/mL in HSE [Table 1] vs. 285 ng/mL in the GP Survey [Table 2]). Participants were distributed relatively evenly across scale scores on the SNDI (range: 8.6%–13.1% in HSE [Table 1] and 8.8%–14.8% in the GP Survey [Table 2]).

**Table 1:** Cotinine by level of nicotine dependence: Health Survey for England, 2000–2021 (*n* = 12 736)

	**Unweighted *n***	**Weighted %**	**Mean saliva cotinine ng/mL (95% CI)**	**Mean cigarettes per day (95% CI)**
Whole sample	12 736	100.0	274 (270% to 277%)	12.9 (12.7% to 13.0%)
Time to first cigarette of the day				
1 ≥2 hours	3128	25.4	129 (124% to 134%)	5.2 (5.0% to 5.4%)
2 1 hour–1 hour 59 minutes	1535	12.0	245 (238% to 252%)	10.6 (10.3% to 10.9%)
3 30–59 minutes	2103	16.9	291 (285% to 298%)	13.2 (12.9% to 13.5%)
4 15–29 minutes	1869	14.3	334 (327% to 341%)	15.2 (14.9% to 15.6%)
5 5–14 minutes	2237	17.0	355 (348% to 361%)	17.1 (16.8% to 17.5%)
6 <5 minutes	1864	14.4	376 (368% to 384%)	20.5 (20.0% to 21.0%)
How easy or difficult to abstain for a whole day				
0 Very easy	2213	18.3	131 (124% to 137%)	5.4 (5.1% to 5.6%)
1 Fairly easy	3188	25.1	239 (239% to 250%)	10.5 (10.3% to 10.7%)
2 Fairly difficult	3154	24.7	302 (302% to 313%)	14.0 (13.7% to 14.2%)
3 Very difficult	4181	31.9	348 (348% to 359%)	18.2 (17.9% to 18.5%)
Short Nicotine Dependence Index score				
1 Lowest	1557	13.1	83 (77% to 88%)	3.1 (2.9% to 3.2%)
2	1261	10.0	170 (162% to 177%)	7.1 (6.8% to 7.4%)
3	1095	8.6	225 (217% to 233%)	9.5 (9.2% to 9.7%)
4	1332	10.6	262 (254% to 270%)	11.4 (11.0% to 11.7%)
5	1516	12.1	298 (290% to 306%)	13.1 (12.8% to 13.5%)
6	1681	13.0	323 (315% to 331%)	15.3 (15.0% to 15.6%)
7	1567	11.9	359 (351% to 367%)	16.7 (16.4% to 17.1%)
8	1479	11.2	368 (359% to 376%)	18.5 (18.1% to 18.8%)
9 Highest	1248	9.6	387 (377% to 396%)	22.1 (21.5% to 22.8%)

**Table 2:** Cotinine by level of nicotine dependence: GP Survey, 1989 (*n* = 1508)

	***n***	**%**	**Mean saliva cotinine ng/mL (95% CI)**	**Mean cigarettes per day (95% CI)**
Whole sample	1508	100.0	285 (275% to 295%)	16.4 (15.9% to 17.0%)
Time to first cigarette of the day				
1 ≥2 hours	330	21.9	124 (110% to 138%)	7.5 (6.9% to 8.2%)
2 1 hour–1 hour 59 minutes	183	12.1	227 (204% to 250%)	12.3 (11.4% to 13.1%)
3 30–59 minutes	211	14.0	265 (243% to 287%)	15.3 (14.2% to 16.4%)
4 15–29 minutes	207	13.7	319 (295% to 344%)	17.4 (16.1% to 18.6%)
5 5–14 minutes	322	21.4	372 (352% to 393%)	20.9 (19.8% to 22.0%)
6 <5 minutes	255	16.9	414 (389% to 439%)	24.9 (23.4% to 26.4%)
How easy or difficult to abstain for a whole day				
0 Very easy	260	17.2	146 (126% to 166%)	7.8 (6.9% to 8.7%)
1 Fairly easy	343	22.7	222 (205% to 239%)	12.4 (11.6% to 13.3%)
2 Fairly difficult	449	29.8	323 (306% to 341%)	17.8 (16.9% to 18.6%)
3 Very difficult	456	30.2	375 (356% to 393%)	22.7 (21.6% to 23.7%)
Short Nicotine Dependence Index score				
1 Lowest	169	11.2	92 (75% to 110%)	5.3 (4.5% to 6.0%)
2	138	9.2	147 (125% to 169%)	8.7 (7.9% to 9.4%)
3	135	9.0	214 (187% to 240%)	11.6 (10.6% to 12.5%)
4	133	8.8	235 (207% to 262%)	12.7 (11.8% to 13.6%)
5	185	12.3	282 (258% to 306%)	16.5 (15.1% to 17.9%)
6	180	11.9	319 (292% to 345%)	17.7 (16.4% to 19.1%)
7	185	12.3	372 (344% to 400%)	19.6 (18.2% to 20.9%)
8	223	14.8	388 (363% to 412%)	22.4 (21.2% to 23.6%)
9 Highest	160	10.6	431 (399% to 463%)	27.4 (25.3% to 29.4%)

**Figure 1:**
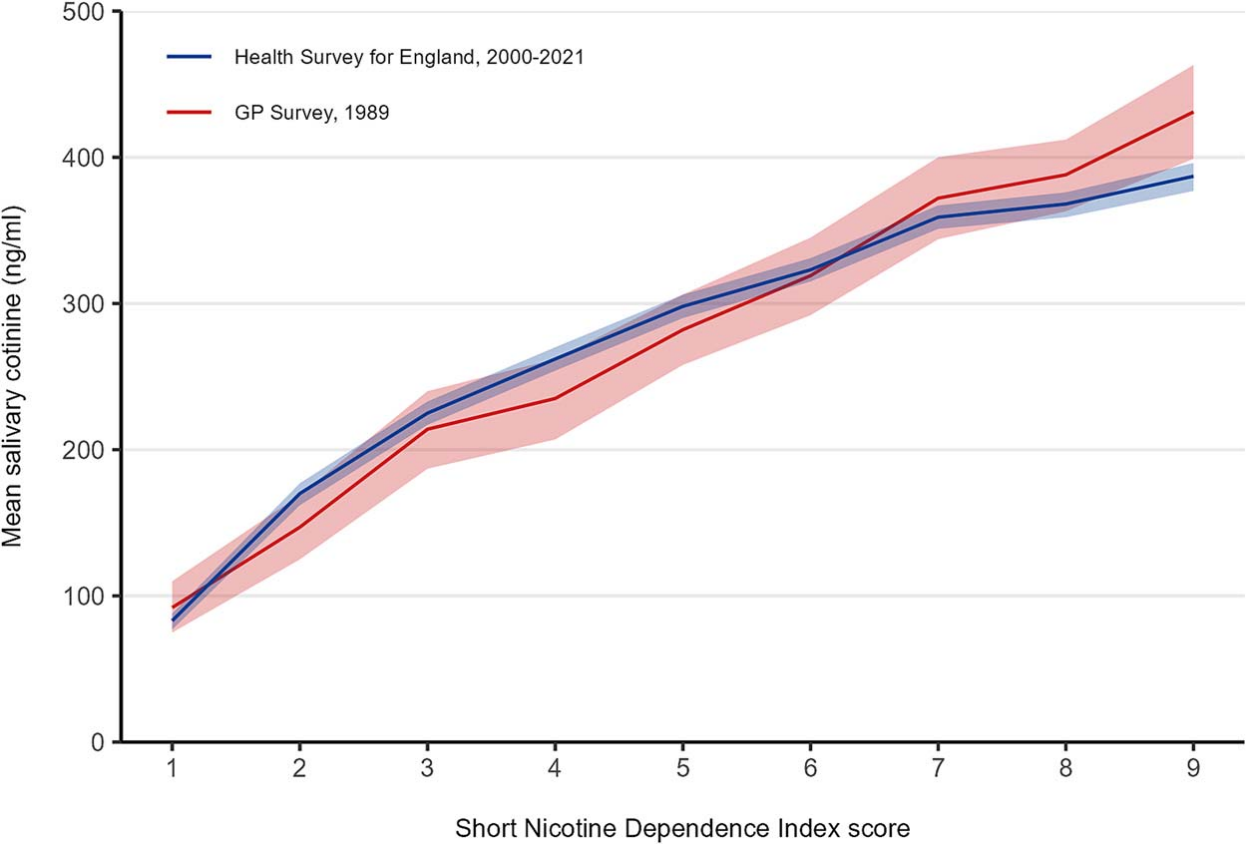
Cotinine by Short Nicotine Dependence Index (SNDI) score. Lines represent mean saliva cotinine in relation to SNDI scores in the Health Survey for England, 2000-2021 (*n* = 12 736) and GP survey, 1989 (*n* = 1508). Shaded bands represent 95% confidence intervals.

**Figure 2:**
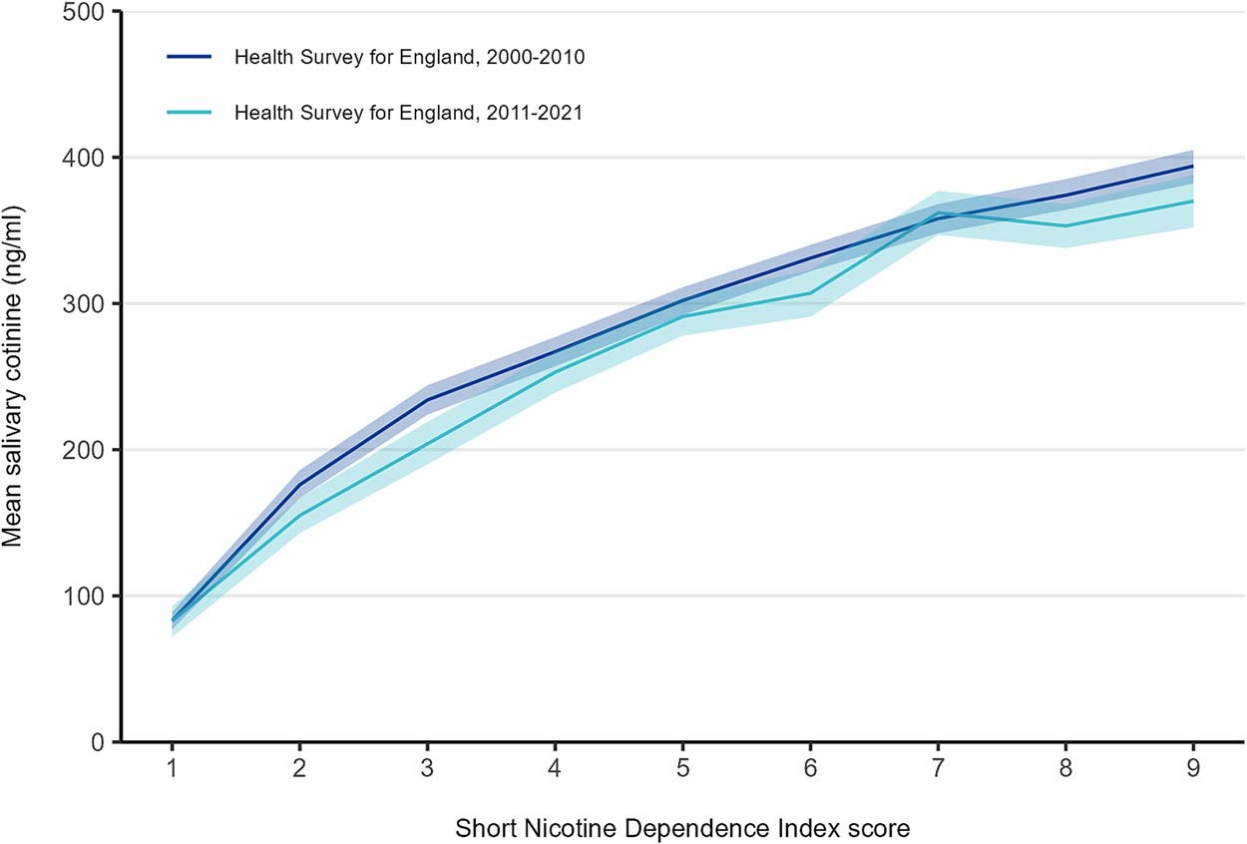
Cotinine by Short Nicotine Dependence Index (SNDI) score in the Health Survey for England, by survey year. Lines represent mean salivary cotinine in relation to SNDI scores in the health survey for England in 2000–2010 (*n* = 8809) and 2011–2021 (*n* = 3927). Shaded bands represent 95% confidence intervals.

Across both datasets, saliva cotinine concentrations increased approximately linearly with scores on the SNDI, demonstrating strong evidence of a positive association between self-reported dependence and nicotine intake. In the HSE sample (Table 1), cotinine ranged from 83 ng/mL among those with the lowest dependence score (1) to 387 ng/mL among those with the highest (9). Mean daily cigarette consumption increased in parallel, from 3.1 to 22.1 cigarettes per day across the same range. A similar gradient was observed in the GP Survey (Table 2), despite higher overall cigarette consumption. Cotinine levels rose from 92 ng/mL at the lowest score to 431 ng/mL at the highest, with cigarettes per day increasing from 5.3 to 27.4. These patterns are clearly illustrated in Fig. 1, with a near-linear rise in cotinine with increasing dependence scores that was virtually identical across the two samples. The pattern was also consistent within HSE when we compared data collected between 2000–2010 and 2011–2021 (Fig. 2). Based on cotinine levels, the estimated daily nicotine intake in the HSE sample ranged from 8 mg among those scoring 1 on the SNDI to 39 mg among those scoring 9. In the GP Survey sample, the corresponding estimates were very similar, at 9 mg and 43 mg.

Both SNDI items showed graded relationships with cotinine and daily cigarette consumption (Tables 1–2). For time to first cigarette, cotinine levels were lowest among those who delayed smoking for ≥2 hours after waking (129 ng/mL in HSE; 124 ng/mL in GP Survey) and highest among those who smoked within 5 minutes (376 ng/mL in HSE; 414 ng/mL in GP Survey). For difficulty abstaining for a day, cotinine levels were markedly higher among those reporting greater difficulty (348 ng/mL in HSE and 375 ng/mL in GP Survey for those finding it “very difficult”) compared with those finding it “very easy” (131 ng/mL in HSE; 146 ng/mL in GP Survey). The alpha for internal consistency of the two items was 0.66 in HSE and 0.68 in the GP Survey.

In both datasets, the SNDI explained a greater proportion of the variance in cotinine than the HSI (30.4% vs. 27.8% in HSE, 28.3% vs. 25.0% in GP Survey; Table 3). The extension of the measure of time to first cigarette from four response options (in the HSI) to six response options (in the SNDI) explained more variance in cotinines, as did the use of the measure of difficulty abstaining rather than cigarettes per day (Table 3).

**Table 3:** *R* ^2^ values from models predicting salivary cotinine using different nicotine dependence measures

**Model**	**HSE, 2000–2021**	**GP Survey, 1989**
**Short Nicotine Dependence Index**		
Item 1: Time to first cigarette of the day	0.274	0.263
Item 2: How easy or difficult to abstain for a whole day	0.199	0.175
Composite score	0.304	0.283
**Heaviness of Smoking Index**		
Item 1: Time to first cigarette of the day	0.238	0.239
Item 2: Cigarettes per day	0.186	0.121
Composite score	0.278	0.250

HSE: Health Survey for England.

Values shown are the *R*^2^ from separate linear regression models predicting salivary cotinine from each item of the Short Nicotine Dependence Index and Heaviness of Smoking Index and their composite scores, all treated as continuous variables.

## Discussion

This study introduces and demonstrates preliminary validity of a brief two-item SNDI that captures two core features of dependence: the urge to smoke (indexed by time to first cigarette) and difficulty abstaining (assessed via self-reported ease of going a day without smoking). Using data from two large population surveys from different time periods and smoking contexts, we found strong and graded associations between the composite score and saliva cotinine, a well-established biomarker of nicotine intake. Cotinine concentrations increased steadily with higher scores, indicating that the scale effectively captures meaningful variation in nicotine dependence across diverse samples.

These findings are consistent with prior research identifying time to first cigarette as the strongest single-item predictor of nicotine intake.^27^ However, our scale extends this predictive value by incorporating difficulty abstaining, a more subjective but equally relevant marker of dependence. Each item independently explained variation in cotinine, and their combination yielded stronger associations than either item alone.

The SNDI outperformed the widely used and validated HSI^6^^,^^28^ in predicting cotinine levels in both datasets. This appears to be due to two key refinements: a more granular time-to-first-cigarette item (six response options instead of four) and the replacement of cigarettes per day with a subjective indicator of difficulty abstaining. These changes allow the new scale to better capture both motivational (internal drive to use nicotine) and behavioral (observable smoking behavior) components of dependence. Notably, the scale performed robustly across three decades, including during periods marked by a rise of non-daily smoking^29^ and the increasing use of non-combustible nicotine products,^18^^,^^20^ supporting its robustness and relevance. Consistent with previous studies,^13–15^ we also found that average cotinine levels remained relatively stable despite major declines in reported cigarette consumption. This suggests that people may be compensating by smoking more intensively (eg, inhaling more deeply or more frequently),^16^^,^^17^ undermining the usefulness of using cigarette count to measure nicotine dependence.

The SNDI’s brevity offers significant practical advantages. Unlike longer tools such as the six-item FTND,^7^ the two-item SNDI is quick to administer and score, making it well-suited for surveillance, clinical screening, and epidemiological research. Its simplicity reduces respondent burden and administrative costs—an important consideration for large-scale studies. In addition, by avoiding reliance on cigarette consumption, the scale may potentially be useful for assessing dependence on other nicotine products (eg, e-cigarettes). This is increasingly relevant in contexts where nicotine use extends beyond daily cigarette smoking to include more non-daily cigarette smoking, non-cigarette tobacco smoking, and use of e-cigarettes, heated tobacco, and nicotine pouches.^18^ For instance, when surveying vaping, the two SNDI items might become “How soon after waking do you usually first use your vape?” and “How easy or difficult would you find it to go without vaping for a whole day?”

Several limitations warrant consideration. The 1989 GP Survey, while valuable for historical comparisons, used a regional rather than nationally representative sample. However, results were consistent with the nationally representative HSE sample, supporting the scale’s generalizability. While saliva cotinine is a reliable biomarker of nicotine intake, it reflects short-term exposure and can be influenced by factors like time since the last cigarette, route of administration, and individual metabolic differences.^30^ The response options for the item assessing time to first cigarette were limited to those included in the 1989 GP Survey and, while they extend the original four-level categorization, it is possible that providing a more detailed list of response options may provide greater sensitivity. This is something that could be explored in future studies to further refine the measure. Finally, we were only able to evaluate the scale in relation to cigarette smoking. Further research is needed to test the scale’s validity among users of alternative nicotine products (amending the item wording, as required) and across demographic subgroups. In addition to assessing associations with cotinine, longitudinal studies are needed to evaluate the scale’s ability to predict the severity of nicotine withdrawal symptoms and cravings during the early weeks of a smoking quit attempt—and, ultimately, to identify who is likely to succeed or relapse.

In conclusion, the SNDI provides an efficient and scalable alternative to traditional dependence measures. Its strong association with cotinine and superior performance compared to existing brief tools supports its use in tobacco research, clinical practice, and public health surveillance—particularly in evolving nicotine use environments where measures relying on typical consumption (which has changed considerably over time) may fall short.

## Supplementary Material

Supplementary_File_ntaf204

## Data Availability

Data are available from the corresponding author.
