# Supplementary material for: The Short Nicotine Dependence Index: A Simple and Versatile Self-Report Measure of Nicotine Dependence for General Populations
Source: Nicotine Tob Res. 2025 Oct 8;28(4):602–8. doi: 10.1093/ntr/ntaf204 (PMC13008583; doi:10.1093/ntr/ntaf204)
Supplement: Supplementary_File_ntaf204 [file supplementary_file_ntaf204.pdf]

Table. Association of each dependence item with cotinine

	B	95% CI
Health Survey for England, 2000-2021		
Time to first cigarette of the day	36.8	34.9-38.8
How easy or difficult to abstain for a whole day	36.7	33.6-39.7
GP Survey, 1989		
Time to first cigarette of the day	33.7	23.9-43.5
How easy or difficult to abstain for a whole day	45.3	39.4-51.1

Data shown are results from a linear regression model predicting cotinine (ng/ml) from the two dependence items that form the SNDI (treated as continuous variables, with higher scores indicating greater dependence).
